# Large-scale genomic analyses reveal the population structure and evolutionary trends of *Streptococcus agalactiae* strains in Brazilian fish farms

**DOI:** 10.1038/s41598-017-13228-z

**Published:** 2017-10-19

**Authors:** Gustavo M. Barony, Guilherme C. Tavares, Felipe L. Pereira, Alex F. Carvalho, Fernanda A. Dorella, Carlos A. G. Leal, Henrique C. P. Figueiredo

**Affiliations:** National Reference Laboratory for Aquatic Animal Diseases (AQUACEN) of Ministry of Agriculture, Livestock and Food Supply, Federal University of Minas Gerais, Belo Horizonte, Minas Gerais Brazil

## Abstract

*Streptococcus agalactiae* is a major pathogen and a hindrance on tilapia farming worldwide. The aims of this work were to analyze the genomic evolution of Brazilian strains of *S. agalactiae* and to establish spatial and temporal relations between strains isolated from different outbreaks of streptococcosis. A total of 39 strains were obtained from outbreaks and their whole genomes were sequenced and annotated for comparative analysis of multilocus sequence typing, genomic similarity and whole genome multilocus sequence typing (wgMLST). The Brazilian strains presented two sequence types, including a newly described ST, and a non-typeable lineage. The use of wgMLST could differentiate each strain in a single clone and was used to establish temporal and geographical correlations among strains. Bayesian phylogenomic analysis suggests that the studied Brazilian population was co-introduced in the country with their host, approximately 60 years ago. Brazilian strains of *S. agalactiae* were shown to be heterogeneous in their genome sequences and were distributed in different regions of the country according to their genotype, which allowed the use of wgMLST analysis to track each outbreak event individually.

## Introduction

*Streptococcus agalactiae* (Lancefield’s group B Streptococcus, GBS) is a Gram-positive coccus that causes septicaemia and meningoencephalitis in many species of marine and freshwater fish worldwide^1–5^. This bacterium may also cause septicaemia and meningitis in human new-borns^6^ and has already been reported in other animals, including guinea pigs, camels, cats, dolphins, horses and frogs^7^. This disease is a major obstacle to the expansion of Brazilian aquaculture because it causes high prevalence in Nile tilapia (*Oreochromis niloticus*), the most frequently farmed fish in Brazil^3^. GBS streptococcosis in tilapia farming occurs mainly in temperatures above 27 °C and it leads to a high economic impact due to high mortality and its ability to evolve quickly^3,5^.

Several genotyping methods have been used to study the population structure of *S. agalactiae* infecting humans and animals, including the evaluation of likelihood of cross-species transmission^8,9^, as capsular serotyping^10^ and multilocus sequence typing (MLST)^11^. The capsular serotyping has been used to discriminate the GBS strains into ten serotypes (Ia, Ib, II-IX)^12^, since each serotype is considered antigenically and structurally unique^7^. The fish GBS isolates are commonly characterized as serotypes Ia, Ib and III^13^. However, the discriminatory potential of this technique has been described as low for evaluation of epidemiological studies^14^. The main epidemiological tool applied in studies of GBS diseases is the MLST^11,15^, which can discriminate strains in lineages (sequence type, ST) and combine them based on genetic proximity in clonal complexes (CC). This method has proven efficient for understanding evolutionary stories between lineages and has so far discriminated fish GBS isolates worldwide in several CCs, including the fish-specific CC552^4,16^. However, MLST cannot fully discriminate many strains from different host species, geographical origins or outbreaks, grouping them into the same sequence type or clonal complex. Examples of this are several strains from human, bovine, feline and rodent sources belonging to ST-103^4,6,17,18^ and ST-7, which groups isolates from fish, humans and dolphins^13^. Also, a previous study revealed that Brazilian fish GBS were almost exclusively from CC552, including ST-552, ST-553 or ST-260^4^ been also this CC predominant in Latin America^19^.

With the advancements in the next-generation high-throughput sequencing and statistical tools based on molecular evolutionary theory, it became possible to perform comparative genome analysis among closely related strains. This strategy had a high resolution, enabling the discrimination of microorganisms at the single nucleotide level, providing thus a new method for typing known as genome-level typing tool^20^. In recent studies of different bacterial species, the use of a larger set (or even all) of alleles from a genome, through the whole-genomes MLST (wgMLST) has shown the possibility to distinguish between closely related strains of a given species even with epidemiological conditions of difficult discrimination^21,22^, as a single outbreak or different anatomic niches in a single patient^17,18^. As such, the wgMLST is emerging as a tool for epidemiological and evolutionary studies and has a promising future in molecular epidemiology of microbial pathogens^23,24^. This kind of information is very strategic in order to apply control measures to avoid he spread of specific bacterial lineages as well as the monitoring of vaccination programs, since the efficacy of *S. agalactiae* vaccine to Nile tilapia seems to be linked to strain specificity^25^. In addition, genome sequencing also allows the elucidation of the temporal and spatial dynamics of evolution of a pathogen through Bayesian phylogenetic methods^26^. This present work aimed to evaluate the population structure and evolutionary trends of the fish pathogen *S. agalactiae* in Brazil and to establish a farm-to-farm approach for the epidemiological tracking of different bacterial clones using genomic methods.

## Results

### Assembly, genomic features and *in silico* serotyping

In order to provide data to perform a large-scale analysis of *S. agalactiae* in Brazil, the 38 strains of the Next Generation Sequence (NGS) project from the National Reference Laboratory for Aquatic Animal Diseases (AQUACEN) culture collection were sequenced. The main features of these 38 genomes along with one previously sequenced strain by our group^27^ are shown in Table 1. The vertical coverage from Ion Torrent sequencing for all sequenced strains of this work (*n* = 38) was 224 ± 88 fold. All isolates were comprised of one single chromosome with an average size of 1,844,131 ± 4,460 bp. The number of coding DNA sequence (CDS) varied from 1,503 to 1,729, the number of pseudogenes varied from 98 to 320 and the average G + C content was 35.48%, for the 39 genomes. A search on the whole-genome sequence was performed to assign the capsular serotype using an in-house script, and all strains were typed as Ib.

**Table 1:** Main genomic features and sequencing type of Brazilian GBS isolates.

Isolate	Size (bp)	CDS^1^	Pseudogenes	# of tRNA	Vertical Coverage (~fold)	Sequence-type
SA01	1841943	1656	172	62	76	NT^2^
SA05	1841945	1652	172	62	207	NT^2^
SA09	1841929	1638	189	62	69	NT^2^
SA16	1841859	1690	129	62	83	NT^2^
SA20^3^	1841952	1678	233	62	1443	NT^2^
SA30	1841729	1592	234	62	163	NT^2^
SA33	1841628	1599	224	62	161	NT^2^
SA53	1848970	1700	123	64	187	260
SA73	1848838	1588	244	64	154	260
SA75	1849016	1667	159	64	233	260
SA79	1841946	1654	171	62	227	NT^2^
SA81	1840363	1687	129	62	240	NT^2^
SA85	1849989	1592	238	64	210	927
SA95	1856590	1707	122	64	304	927
SA97	1856410	1580	260	64	158	927
SA102	1849521	1510	320	64	135	927
SA132	1852032	1657	170	64	344	260
SA136	1849103	1643	180	64	377	260
SA159	1841483	1503	318	62	130	NT^2^
SA184	1841893	1608	218	62	218	NT^2^
SA191	1848676	1599	232	64	157	260
SA195	1841715	1596	232	62	175	NT^2^
SA201	1841834	1691	144	62	239	NT^2^
SA209	1841835	1684	141	62	268	NT^2^
SA212	1841962	1683	141	62	256	NT^2^
SA218	1849985	1706	121	64	269	927
SA220	1841963	1672	149	62	188	NT^2^
SA245	1848955	1690	135	64	318	260
SA256	1848972	1687	138	64	206	260
SA289	1848987	1705	122	64	235	260
SA330	1842081	1694	132	62	222	NT^2^
SA333	1842037	1705	119	62	226	NT^2^
SA341	1842113	1702	129	62	322	NT^2^
SA343	1841977	1702	118	62	464	NT^2^
SA346	1841984	1698	122	62	424	NT^2^
SA374	1842255	1644	184	62	162	NT^2^
SA375	1842219	1684	149	62	192	NT^2^
SA623	1842115	1729	98	64	134	NT^2^
SA627	1842066	1729	99	64	227	NT^2^

^1^Only undisrupted protein coding sequences, without pseudogenes.

^2^Non-typeable.

^3^Strain features from Pereira *et al*.^20^.

### MLST and eBURST profiles

**Figure 1:**
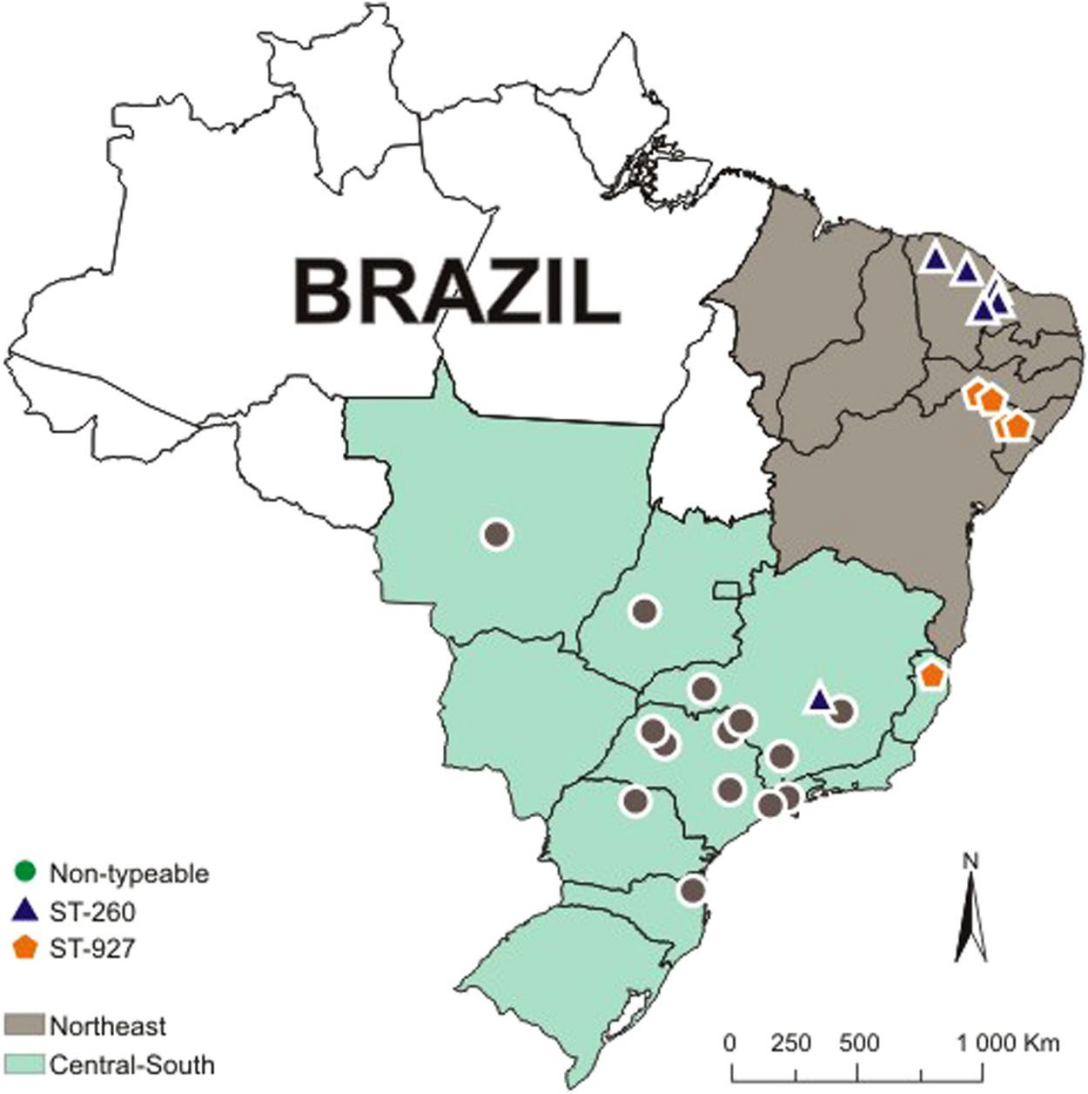
Map of the distribution of the *S. agalactiae* sequencing types throughout Brazil, generated in the OpenJump software version 1.6.3^43^.

**Figure 2:**
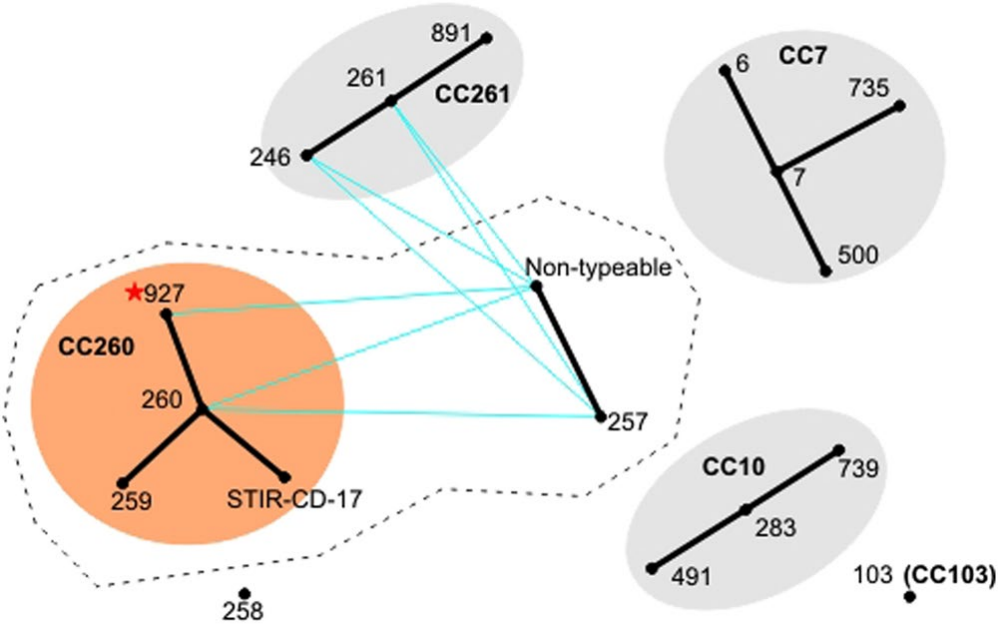
Reconstruction of evolutionary relationships between piscine *S. agalactiae* through eBURST analysis from MLST. Points represent STs, circles represent clonal complexes, black lines represent single-locus variants, blue lines represent double-locus variants and isolated points represent singletons. eBurst groups arising exclusively from piscine strains are CC260, CC261 and the group formed by ST-257 and non-typeable strains. CC10 and CC7 arise from piscine and human strains. The dashed area delineates the previously known CC552.

From the whole genomes sequenced, the ST and clonal complexes were reconstructed in order to compare the population structure of Brazilian GBS strains with other GBS fish strains isolated in other countries. The 39 strains presented two distinct sequence types: one previously reported in Brazil (ST-260) plus a new ST that was submitted to PubMLST and named ST-927 (Table 1, Fig. 1). Additionally, several strains (*n* = 25) were non-typeable due to a partial deletion in the *glcK* locus and the consequential hindrance of allelic typing, an event previously described by Assis, *et al*.^28^. The eBURST analysis of all piscine-related STs is shown in Fig. 2. This analysis grouped the strains of three previously established CCs, as follows: CC10 with ST-283, ST-491 and ST-739; CC7 with ST-6, ST-7, ST-500 and ST-735; and CC261 with ST-246, ST-261 and ST-891. In addition, it established two newly proposed eBurst groups: the CC260 with ST-260 as a founder, ST-259, and the newly ST-927, and the group comprised of ST-257 and the non-typeable strains with a SLV, a close genetic relation. Strain STIR-CD-17 (GenBank accession number ALXB01) has no established ST, but its ST is different from ST-260 by a single nucleotide polymorphism at *tkt*. Neither ST-103 nor ST-258 shared enough alleles with any of the other piscine strains to be considered as genetically related.

### Genomic similarity

**Figure 3:**
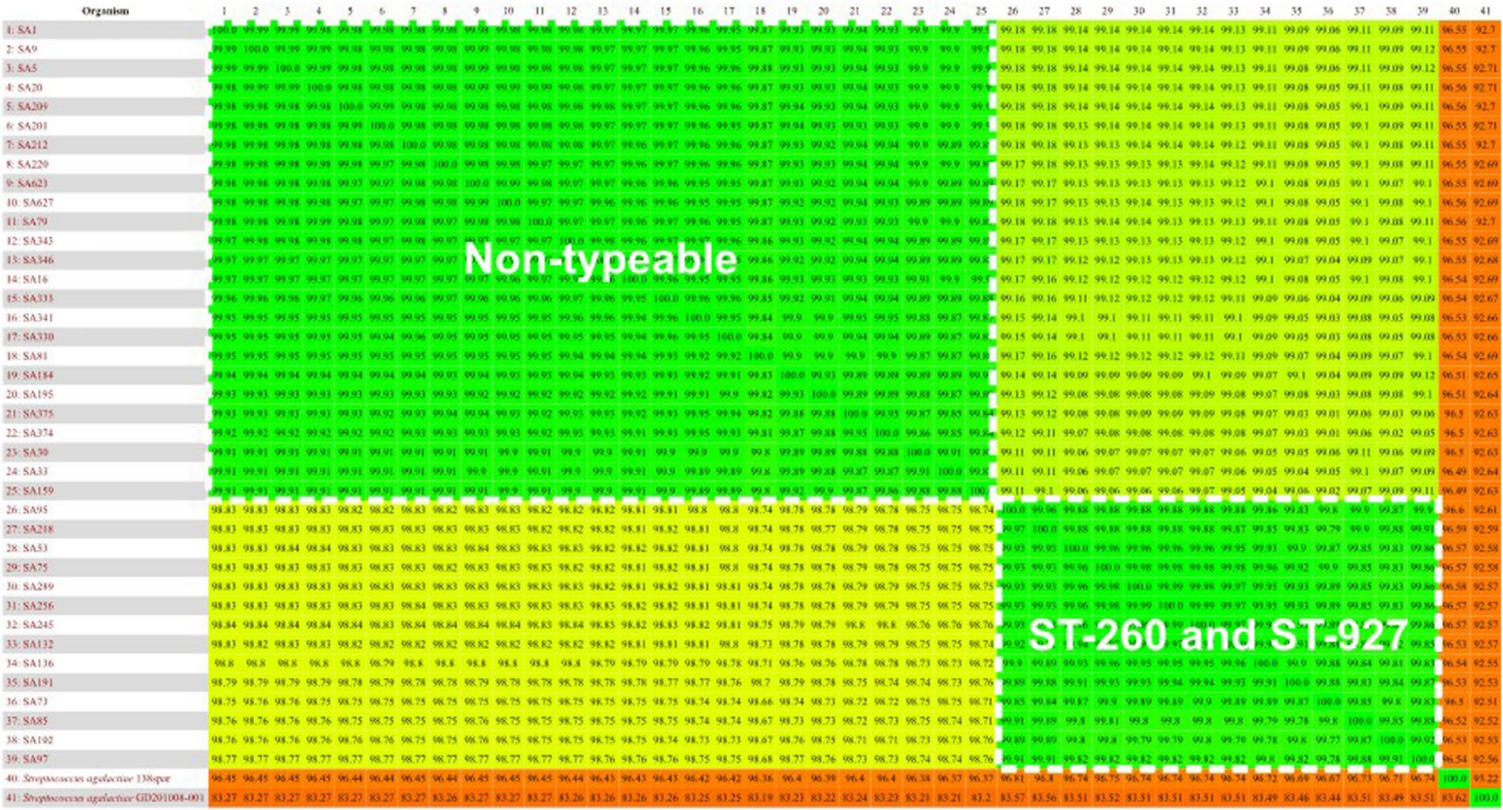
All against all average nucleotide-blast identity from similarity comparison between the studied genomes. Increasing in similarity from red to green. Areas delimited by dashes harbour intra-ST similarities.

Considering the low level of ST diversity observed in the Brazilian GBS fish strains, the 39 genomes were submitted to Gegennes, whose strategy is the comparison of similarities of whole genome contents through Blastn percentage of identity. The all versus all analysis of genomic similarity is shown in Fig. 3. All the studied piscine *S. agalactiae* strains showed high similarity (>98%), but two groups were slightly distinct. One was composed of MLST non-typeable isolates, and the other strains ST-260 plus ST-927, showed higher similarities within groups rather than between groups.

### Phylogenomic analysis based on wgMLST

**Figure 4:**
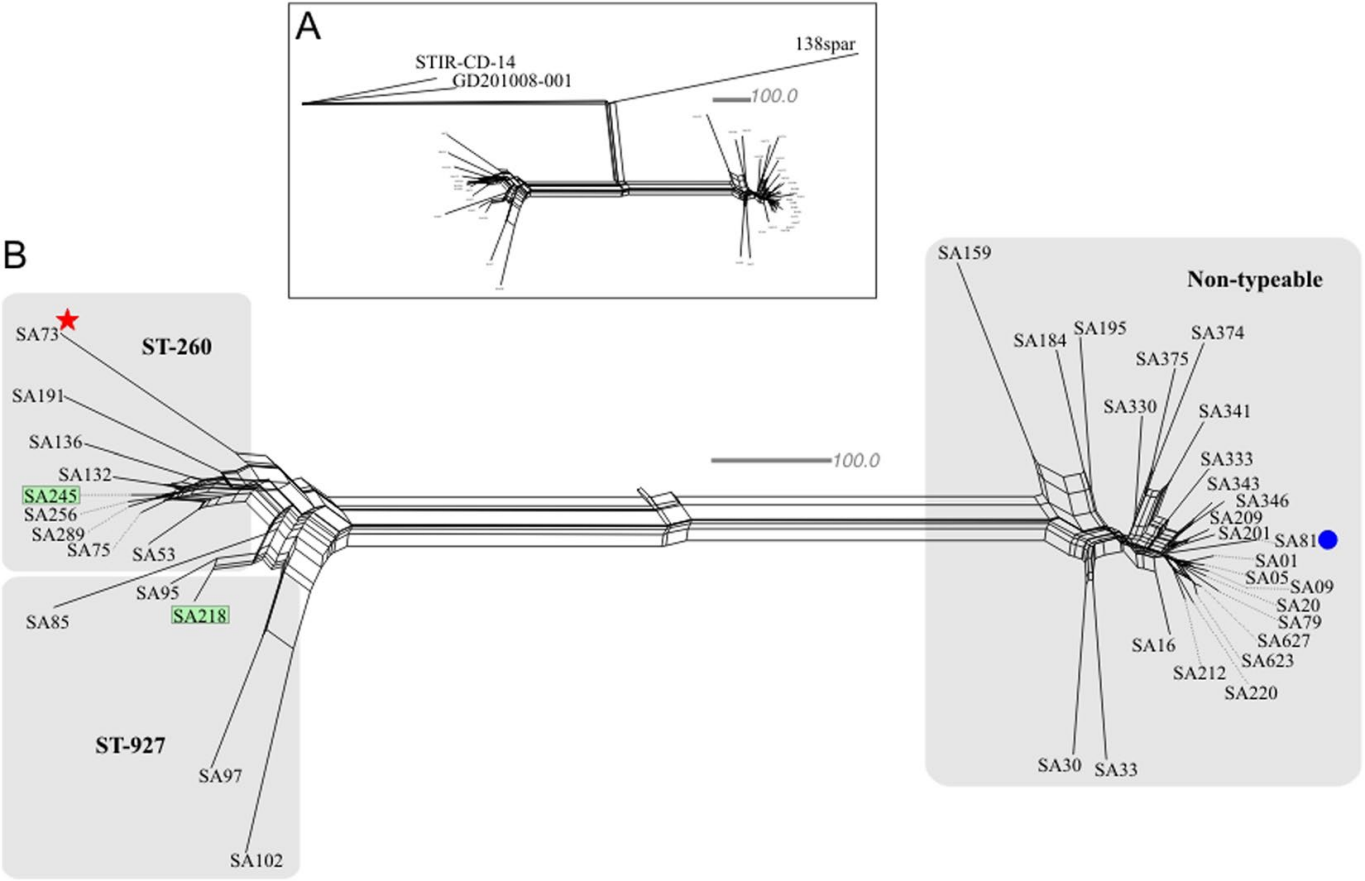
Phylogenomic NeighborNet network of wgMLST data. Scale bar measures 100 different alleles. (**A**) The four major phylogenomic groups, with a focus on Brazilian strains. (**B**) Zoom of the Brazilian phylogenomic splits. The left side of the network harbours all ST-927 and ST-260 strains, all from grow-out farms with the exception of the SA73 (marked with a star), which is from a hatchery. All strains from those two STs are from the Northeast region with the exception of the marked isolates SA218 and SA245, which both emerged from the Southeast region. The right side of the network harbours all non-typeable strains, which all arise from the Central-South macroregion. The strain SA81 (marked with a circle) was obtained from a diseased catfish, while the remaining strains were obtained from Nile tilapia.

As genomic similarities did not improve discriminatory results after using pre-genomic MLST based techniques, a survey of high-resolution tools was conducted. Thus the wgMLST technique, which is known to provide a scalable means to study the whole-genome variation^29^, was designated to this study. The NeighborNet phylogenomic network separated all isolates into four main groups based on the “All_loci” comparison (Fig. 4A). One group was composed by the single strain 138spar (ST-261, USA) and the other group included the strains STIR-CD-14 (ST-491, Vietnam) and GD201008-001 (ST-7, China). The Brazilian strains were divided in two groups (Fig. 4B), with one group formed by all non-typeable strains (all arising from the Central-South region) and the other major group formed by ST-260 and ST-927 strains (arising mainly from the Northeast region).

### Bayesian analysis of evolution and emergence of the Brazilian *S. agalactiae* clade

**Figure 5:**
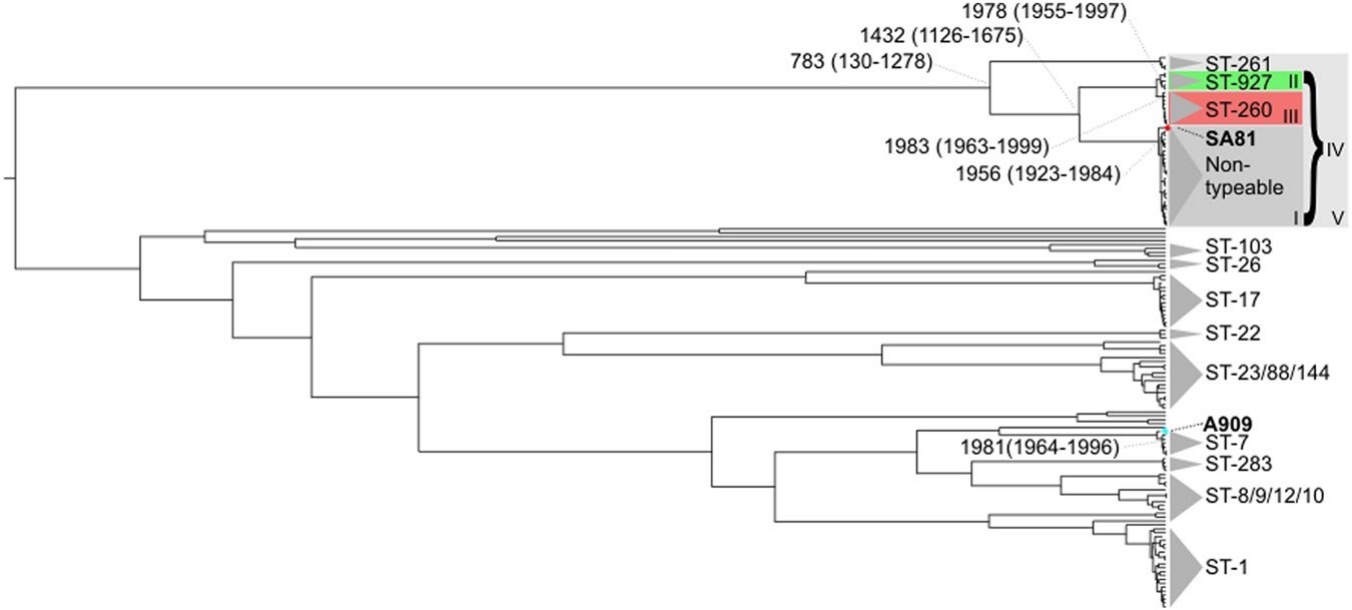
Bayesian evolutionary analysis of *S. agalactiae*. The upper branch of the tree shows the origin of taxa IV, which contains Brazilian piscine isolates of *S. agalactiae* and taxa V, which is taxa IV in addition to ST-261. The marked strain SA81 is an exclusive Brazilian catfish isolate, while all the other Brazilian isolates are from tilapia. The time to tMRCA of each taxa of interest is provided and marked with a grey dashed line. The years between brackets are the 95% highest probability density. On the lower region of the tree, within ST-7, the marked strain A909 was isolated from human, while the other ST-7 isolates were from fish.

In order to evaluate the evolutionary trends of the population of Brazilian GBS fish strains, particularly the temporal emergence of different clones, a Bayesian analysis was performed based on substitutions found in the core genome of *S. agalactiae*. The substitution rate and evolution analysis showed a highly convergence with ST grouping of MLST technique (Fig. 5). The analysis was performed using 100 million generations that showed to be an Effective Sample Size (ESS) with values >=200 for key parameters (output variables). The substitution rate of this population was 6.21 × 10^−7^ substitution/site/year (95% highest probability density [HPD], 3.42 × 10^−7^ to 8.97 × 10^−7^). One clade, named taxa V, was formed by the ST-261, whose strains came from the USA, Israel and China and along with ST-927, ST-260 and the non-typeable strains of this work, they seemed to have emerged 1,233 years ago (95% HPD, 738 to 1886), whereas Taxa IV, containing the reported STs of *S. agalactiae* from fish in Brazil, seemed to have emerged approximately 585 years ago (95% HPD, 341 to 890). A third group (Taxa III) was composed only of the ST-260 from Brazilian isolates (although this ST also occurs outside Brazil) and had a time to most recent common ancestor (tMRCA) of approximately 33 years ago, in 1983 (95% HPD, 1963 to 1999). Finally, two groups exclusively from the Brazilian samples (Taxa II – non-typeable strains and Taxa I – ST-927) emerged respectively, in 1956 (95% HPD, 1923 to 1984) and in 1978 (95% HPD, 1955 to 1997).

## Discussion

MLST is the main epidemiological tool for studying human and animal diseases caused by *S. agalactiae*, and it has been widely used to study evolutionary relationships between strains from different epidemiological sources, such as hosts species, geographical distances, and periods of isolation^4,9,13,30–32^. Nevertheless, it is already known that by using only seven genetic loci might result in the neglect of some genetic information, such as lateral genetic transfer events or phage-related genes, which compromises the resolution of the molecular characterization^33^. Alternatively, the use of genomic tools would be suitable to find divergence in close related GBS strains.

The MLST utilizes seven genes that play important cellular functions^11,34^ and whose mutations could possibly be subject to purifying selection. The studied strains could be discriminated into two STs and one non-typeable group. A previous study^4^, had described the ST-552 and ST-553, however theses STs were composed only by strains identified as non-typeable that shared a partial gene deletion event on *glcK* gene, as described by Assis *et al*.^28^. A PCR reaction targeting the *glcK* gene were performed for all Brazilian strains based on Godoy *et al*.^4^, and as a result all PCRs were found to be negative using electrophoresis on a 1.5% agarose gel (data not shown). This discovery resulted in the deconstruction of the previously described CC552^4^, which were comprised of ST-257, ST-259, ST-260, ST-552 and ST-553. Therefore, it was then established two eBurst groups, as follows: firstly the CC260 group, comprising ST-260 (founder, which formerly comprised CC552), ST-927 (newly described), ST-259 (which formerly comprised CC552), and the strain STIR-CD-17; and secondly, the group comprised of ST-257 (which cannot be considered as a CC since it does not have a founder; it was formerly comprised of CC552) and the non-typeable strains. These two eBurst groups and the CC261 had a similar MLST profile, since they shared five identical alleles, suggesting an evolutionary relationship.

In the present work, the three obtained MLST groups (two STs and the non-typeable strains) presented a geographical distribution pattern, with the non-typeable strains occurring exclusively in the Central-South region and the ST-260 and ST-927, occurring mainly in the Northeast, which is in accordance with a previous study^4^. The occurrence of ST-260 and ST-927 (SA245 and SA218, respectively) in the Southeast region shows that these two types have been circulating through the country, which is not surprising, as *S. agalactiae* is believed to be transmitted by contact between animals^2,3^.

Genomic similarity results (Fig. 3) showed that each ST comprises strains with similar sequences (>99.9%), and that the differences between the STs were small (<2%). It has been previously shown through comparative genomics that *S. agalactiae* has a genome structured with a stable “back-bone” and that other elements are responsible for differences between many lineages^17^. Therefore, small differences between genomes probably occur due to polymorphisms in the gene sequences. Fish-associated GBS isolates are known to harbour some genetic content that is not shared with other CCs^16^ and that may be associated with host adaptation. However, there is no information regarding genomic diversity in a population of closely related *S. agalactiae* strains that could be used to track every source of an outbreak of streptococcosis. The genomic features of the Brazilian isolates of *S. agalactiae* showed a number of pseudogenes varying from 98 to 320 between isolates and even varied inside each genomic lineage. It is known that piscine GBS strains are going through a reductive evolution, and a high percentage of pseudogenes are commonly seen, over 10% of the genome, which is believed to be an adaptative strategy of these hosts^34^. The analysis of genomic similarities corroborated with the MLST results, though it was not was sufficient to discriminate the closely related strains (Fig. 3).

Thus, a wgMLST was performed to depict the population structure of *S. agalactiae* isolated from fish in Brazil. According to Maiden, *et al*.^21^, the wgMLST has the potential to discriminate very closely related strains or single clone pathogens. These results strengthened the previous comparative analyses, since the phylogenomic network based on all loci comparisons revealed that the genomic types were similar. The geographical discrimination of the isolates was in accordance with the MLST, but by the use of the wgMLST approach it was possible to observe the relative genetic distances between each strain. The closest contemporary strains (SA623 and SA627, both isolated in 2015) diverged by 22 alleles, suggesting that each analyzed Brazilian isolate belongs to a single clone.

As shown in Fig. 4, in the ST-927 group, the two strains from the state of Pernambuco (SA102 and SA97) were closely related, and the same was seen for the strains from the Alagoas state (SA85 and SA95). However, in the ST-927 (mainly from the Northeast region), the strain SA218 from the Southeast region was most closely related to SA95, and in the ST-260 (mainly from the Northeast region), the SA245 from the Southeast region was most closely related to SA256, reinforcing the assumption of pathogen transmission by infected fish dislocations, which was raised after the MLST analysis, even with the interregional trade.

Moreover, in the non-typeable group, the lower axis contained the older isolates, while the upper axis contained the newest ones and showed that more contemporary strains had fewer allelic differences, suggesting that mutations were occurring through a temporal scale. Inside this group, the genome of the catfish isolate (SA81) was very closely related to some tilapia isolates (Fig. 4B). Currently, tilapia is the main piscine host for *S. agalactiae* in Brazil. Nevertheless, the emergence of new Brazilian fish species as potential hosts for this pathogen suggests that the host range might be even broader (tilapia and the parental catfish species of the hybrid catfish belong to different taxonomic orders) and it represents a great potential hazard for the Brazilian aquaculture.

Based on the Bayesian evolutionary tree (Fig. 5), all piscine GBS strains from Brazil have emerged from a single branch and are grouped in accordance with the MLST. This group emerged approximately 585 years ago, which is more recent than the group composed by other American, Chinese and Israeli piscine *S. agalactiae* strains from ST-261 and that they emerged approximately 1,234 years ago. It is evident that since the divergence from ST-261, the group composed of ST-260, ST-927 and the non-typeable strains are evolving more rapidly and further diversifying (intra-groups I, II and III in Fig. 5). These results showed that the main ST that nowadays occurs in Brazil had emerged quite recently (apart from 1956, for non-typeable strains) and, together with the genetic diversity depicted by the wg-MLST, might represent a concern for the development and use of vaccines with broad protection and the possibility of adaptation to new hosts. Inside the non-typeable group, SA81 (marked with a circle in Fig. 5), isolated from catfish, emerged from a different branch of the GBS strains isolated from tilapia, which suggests that microevolution is occurring within this group that is leading to different host tropisms. However, the genetic basis of this possible adaptation needs to be evaluated.

Similarly, the human ST-7 strain A909 isolated in 1975 (GenBank accession number CP000114, marked with a star in Fig. 5) emerged previously from a single branch contrast the other six ST-7 strains that were isolated between 2009–2015 (Fig. 5, Supplementary Table 2), which shared a single branch, arose from tilapia host, and emerged contemporaneously with the Brazilian strains in 1981 (1964–1996). Since the emergence of the ST-7, there has been a diversification to different host tropisms, which is in line with previous genomic studies that revealed a close genomic relationship between these isolates^34,35^. Although the evolutionary data pointed to a very recent emergence of new GBS STs, it should be considered that this analysis has some limitations, since other genetics events, such as horizontal gene transfer, are not evaluated by this method.

This study revealed that the Brazilian *S. agalactiae* population of piscine isolates is diverse, spatially distributed according to their sequence type and that many recent evolutionary events are leading the creation of new groups. Furthermore, the study revealed that there are contemporary events leading to the increased diversity of piscine *S. agalactiae* in Brazil, probably evolving along with the expansion of aquaculture in the last five decades. The gene-by-gene approach is a powerful tool that allows us to track outbreaks of streptococcosis by *S. agalactiae* farm-to-farm and to establish spatial links between disease events.

## Methods

### Bacterial strains

The strains of *S. agalactiae* were selected from the AQUACEN culture collection. All strains were previously characterized^4,36^, and its geographic origin and year of isolation of each strain is presented in the Supplementary Table 1.

### DNA extraction and sequencing

For DNA extraction, strains were thawed and grown in blood agar at 28 °C for 48 h. Colonies were collected and submitted to the Maxwell® 16 MDx Research Instrument (Promega, USA) following the manufacturer’s instructions. Genomic DNA were quantified using a Qubit 2.0 Fluorometer (Life Technologies, Thermo Scientific, USA). The strain SA20^37^ was re-sequenced by our group^27^ on a MiSeq sequencer (Illumina, USA) using a Nextera™ DNA Library Prep Kit. All other strains were sequenced on an Ion Torrent Personal Genome Machine™ (PGM) (Life Technologies) with the Ion PGM Sequencing 200 bp Kit following the manufacturer’s recommendations.

### Genomes assembly and annotation

The quality of all raw sequenced data was analysed using FastQC 0.11.1^38^, and an *in-house* script (https://www.github.com/aquacen/fast_sample) was used to obtain reads with a PHRED quality score of at least 20 (i.e., -q 20 parameter) and exclude adaptors sequence (i.e., -l 17 parameter). Genomes were then *de novo* assembled using Newbler 2.0 (Roche, USA) with -urt -noace -m -a 50 parameters, and scaffolds were generated using CONTIGuator^39^, using SA20 as a reference strain and default parameters. Gap filling was performed manually using CLC Genomics Workbench 7.0 (CLC-gw) (Qiagen, USA), mapping the reads to the genomes and gradually extending the flanks of the gaps. Annotation was performed manually for SA20 using Uniprot database (http://www.uniprot.org), which was then used to annotate the other strains by the software Prokka version 1.11^40^, with modification to use nested databases in this order: manually curated CDSs from SA20, RefSeq database only with *S. agalactiae* proteins, and finally all proteins from RefSeq. The annotated genomes visualization and the manual correction of frameshifts were performed on Artemis^41^ and CLC-gw, respectively.

### *In silico* capsular serotyping

An *in-house* script was developed (available at https://www.github.com/aquacen/serotype_Sagalactiae) to automate the protocol designed by Sheppard, *et al*.^42^, to access the capsular serotype of *S. agalactiae* isolates through the whole genome sequence. In summary, this script uses the complete genome sequence of each genome as query in Blastn algorithm, against a database containing the sequences proposed by Sheppard *et al*.^42^, with e-value 1e-100, query coverage >=90% and percentage of identity >=95%.

### MLST genotyping and eBURST analysis

All seven of the MLST loci *adhP* (alcohol dehydrogenase), *pheS* (phenylalanyl-tRNA ligase subunit alpha), *atr* (amino acid ABC transporter), *glnA* (glutamine synthetase), *sdhA* (L-serine dehydratase subunit alpha), *glcK* (glucokinase) and *tkt* (transketolase) were selected from the 39 GBS genomes and then STs were obtained by an *in-house* script (available at https://www.github.com/aquacen/mlst_Sagalactiae). This script searches designated alleles deposited on pubMLST in the genomes through nucleotide-Blast alignment. Moreover, one strain from each ST previously related on fish was selected from the pubMLST and GenBank databases, and all data were submitted to the eBURST algorithm^43^. Clonal complexes were defined using SLV bias with default parameters. The map with the strains geographic location was generated in the OpenJump software version 1.6.3^44^, using Brazilian geographic data available at Brazilian Ministry of Environment web site (http://mapas.mma.gov.br/i3geo/datadownload.htm) and isolation coordinates obtained in the AQUACEN culture collection metadata.

### Genomic similarity percentage analysis

The similarity of whole genomes were compared using Gegenees software version 2.0^45^ along with other piscine *S. agalactiae* strains that had complete genome sequences, 138spar (ST-261) and GD201008-001 (ST-7) (GenBank access numbers CP007565 and CP003810, respectively), to obtain a percentage identity matrix. This matrix was then used to build a similarity heatmap. The parameters set in Genenees were a Fragment size = 200 bp, Step size = 100 bp and a threshold in heat settings of 0%.

### Phylogenomic analysis based on wgMLST

The complete genome sequences of the 39 presented strains together with 138spar and GD201008-001, in addition to the draft genome sequence of ST-491 *S. agalactiae* strain STIR-CD-14 (GenBank access number ANEJ01), were submitted to Bacterial Isolate Genome Sequence Database (BIGSdb). All loci were compared one by one between isolates using a gene-by-gene approach^21^ on a GenomeComparator plugin. All loci scheme was previously generated using all genes cluster from a cd-hit-est software v4.6^46^, with a multi-fasta file containing all predicted genes from all strains (*n* = 42) and the default parameters. A distance matrix with the relative genomic divergence between all isolates was obtained and used to construct a phylogenomic NeighborNet network using SplitsTree 4.0^47^.

### Bayesian estimation of clonal emergence

The 39 strains from this study, along with the *S. agalactiae* genomes available on GenBank that were at least at the scaffold stage with a number of scaffolds >=30 (*n* = 103), were used to perform the substitution rate and Bayesian analyses. Using a highly stringent nucleotide-Blast search with an e-value of 1e-20, the CDS of SA20 were aligned against all other strains. All CDSs that met the following requirements were retained: minimal percentage of identity of 98%, difference of length between query and subject <=5 bp, lacking paralogous sequences, and present in all genomes (*n* = 382 genes) were extracted and concatenated to form the core genome of the species. The core genome of the 142 strains was aligned using MAFFT v7.302b^48^ with the parameter “–auto” enabled. The BEAUti package of BEAST v1.8.3^49^ was used to generate an xml file to run a Bayesian phylogenetic analysis with the following parameters: output files of MAFFT as data, five taxa groups (Taxa I – all the MLST non-typeable strains of this work; Taxa II – all strains of ST-927; Taxa III – all strains of ST-260; Taxa IV – all strains of group I, II, III; and Taxa V – all strains of group I, II, III and ST-261); tip dates (year of isolation, for all fish strains, and year precision, for some strains that lack year of isolation), as described in the Supplementary Table 2, to calibrate the clock rate; general time-reversible (GTR) model with gamma correction plus invariant sites; strict clock; and coalescent exponential growth tree. Markov Chain Monte Carlo (MCMC) was performed using BEAST with 100 million generations and log collection every 100 generations. The Tracer package of BEAST was used to analyze the log collection with a burn-in value of 10% of generations. TreeAnnotator of BEAST was used to output the maximum clade credibility (MCC) tree as a nexus file, and TempEst software v1.5^50^ was used to show the tree previously generated by TreeAnnotator.

BEAST implements strict, lognormal-relaxed, and exponential-relaxed molecular clock models and coalescent constant, exponential and expansion growths, and Bayesian skyline tree models. Furthermore, several substitution models for nucleotides are available. The clock and substitution models combinations were tested with MCMC as described above. Models of Bayesian skyline tree, lognormal-relaxed molecular clock and exponential-relaxed molecular clock, that failed to perform the MCMC algorithm, were discarded. Only results with ESS values greater than 200 for all key parameters were compared. The used model described above had been chosen by the substitution rate HPD cover described in a previous work with the *Streptococcus* genus^51^.

## Electronic supplementary material

Supplementary Information Table 1 and 2
