# Supplementary material for: Large-scale genomic analyses reveal the population structure and evolutionary trends of Streptococcus agalactiae strains in Brazilian fish farms
Source: Sci Rep. 2017 Oct 19;7:13538. doi: 10.1038/s41598-017-13228-z (PMC5648781; doi:10.1038/s41598-017-13228-z)
Supplement: Supplementary file 1 — Supplementary Information Table 1 and 2 [file 41598_2017_13228_MOESM1_ESM.pdf]

Large-scale genomic analyses reveal the population structure and evolutionary trends of *Streptococcus agalactiae* strains in Brazilian fish farms

Gustavo M Barony[1], Guilherme C Tavares[1], Felipe L Pereira[1], Alex F Carvalho[1], Fernanda A Dorella[1], Carlos A G Leal[1], Henrique C P Figueiredo[1*]

Supplementary Table 1. Geographical origin of the 39 strains of *S. agalactiae* isolated from outbreaks of streptococcosis in Brazil

Isolate	City	State	Region	Year of isolation
SA01	Esmeraldas	MG	Southeast	2003
SA05	Linhares	ES	Southeast	2003
SA09	Linhares	ES	Southeast	2005
SA16	Tietê	SP	Southeast	2006
SA20	Itambaracá	PR	South	2006
SA30	Tietê	SP	Southeast	2006
SA33	Zacarias	SP	Southeast	2006
SA53	Jaibaras	CE	Northeast	2007
SA73	Russas	CE	Northeast	2008
SA75	Jaguaribara	CE	Northeast	2008
SA79	Joinville	SC	South	2009
SA81	Lucas do Rio Verde	MT	Central-West	2009
SA85	Piranhas	AL	Northeast	2010
SA95	Piranhas	AL	Northeast	2010
SA97	Jatobá	PE	Northeast	2010
SA102	Petrolândia	PE	Northeast	2010
SA132	General Sampaio	CE	Northeast	2011
SA136	Jaguaribara	CE	Northeast	2011
SA159	Alfenas	MG	Southeast	2011
SA184	Pinheiros	ES	Southeast	2011
SA191	Jaguaruana	CE	Northeast	2011
SA195	São José dos Campos	SP	Southeast	2011
SA201	Linhares	ES	Southeast	2012
SA209	Buritana	SP	Southeast	2012
SA212	Rifaina	SP	Southeast	2012
SA218	Linhares	ES	Southeast	2012
SA220	Rifaina	SP	Southeast	2013
SA245	Betim	MG	Southeast	2013
SA256	Jaguaribara	CE	Northeast	2013
SA289	Jaguaribara	CE	Northeast	2013
SA330	Franca	SP	Southeast	2013

SA333	Goiás	GO	Central-West	2014
SA341	Alfenas	MG	Southeast	2014
SA343	Alfenas	MG	Southeast	2014
SA346	Alfenas	MG	Southeast	2014
SA374	São Paulo	SP	Southeast	2014
SA375	São Paulo	SP	Southeast	2014
SA623	Uberlândia	MG	Southeast	2015
SA627	Uberlândia	MG	Southeast	2015

MG: Minas Gerais; ES: Espírito Santo; SP: São Paulo; PR: Paraná; CE: Ceará; SC: Santa Catarina; MT: Mato Grosso; AL: Alagoas; PE: Pernambuco; GO: Goiás.

Supplementary Table 2. Isolation dates of the strains to the Bayesian phylogenomic analysis [1].

ST	Strain	Year of isolation	Year precision[1]	BioProject Accession Number
1	09mas018883	2003	10	PRJEB1693
1	BG_NI_011	2010		PRJEB2589
1	CZ_NI_004	2008		PRJEB2589
1	CZ_NI_006	2008		PRJEB2589
1	CZ_NI_008	2008		PRJEB2589
1	CZ_NI_009	2008		PRJEB2589
1	CZ_NI_013	2009		PRJEB2589
1	CZ_NI_015	2009		PRJEB2589
1	DE_NI_001	2007		PRJEB2589
1	DK_NI_012	2010		PRJEB2589
1	DK_NI_013	2010		PRJEB2589
1	DK_NI_022	2011		PRJEB2589
1	GB_NI_009	2010		PRJEB2589
1	GB_NI_010	2010		PRJEB2589
1	GBS_ST_1	2015		PRJNA296923
1	IT_NI_0031	2010		PRJEB2589
1	IT_NI_028	2009		PRJEB2589
1	MRI_Z1_212	2002	10	PRJNA86409
1	RBH05	2008		PRJEB2589
1	SS1	1992		PRJNA274384
3	DE_NI_022	2009		PRJEB2589
7	A909	1975	5	PRJNA326
7	GD201008	2010		PRJNA169338
7	GX064	2011		PRJNA258307
7	HN016	2011		PRJNA258321
7	WC1535	2015		PRJNA328400
7	YM001	2011		PRJNA258349
7	ZQ0910	2009	2	PRJNA158929
8	BE_NI_005	2010		PRJEB2589
8	PSS_7736	2005	10	PRJNA254657
9	DK_NI_008	2009		PRJEB2589
10	DE_NI_004	2008		PRJEB2589
10	DK_NI_015	2008		PRJEB2589
12	BG_NI_007	2009		PRJEB2589
12	BG_NI_010	2009		PRJEB2589
17	BG_NI_002	2009		PRJEB2589
17	COH1	1985	5	PRJEB5232
17	DE_NI_013	2009		PRJEB2589
17	DE_NI_036	2010		PRJEB2589

17	DE_NI_037	2010		PRJEB2589
17	DK_NI_001	2009		PRJEB2589
17	DK_NI_007	2009		PRJEB2589
17	GB_NI_003	2009		PRJEB2589
17	GB_NI_004	2010		PRJEB2589
17	IT_NI_009	2008		PRJEB2589
17	NGBS128	2010		PRJNA293561
22	GBS1_NY	2012		PRJNA243853
22	GBS2_NM	2012		PRJNA243854
22	GBS6	2009		PRJNA244773
23	759_SAGA	2013		PRJNA267549
23	BE_NI_001	2009		PRJEB2589
23	BG_NI_004	2010		PRJEB2589
23	BG_NI_005	2009		PRJEB2589
23	CZ_NI_001	2008		PRJEB2589
23	CZ_NI_005	2008		PRJEB2589
23	DE_NI_014	2009		PRJEB2589
23	DE_NI_033	2010		PRJEB2589
23	DE_NI_040	2010		PRJEB2589
23	DK_NI_002	2009		PRJEB2589
23	DK_NI_005	2009		PRJEB2589
23	GB_NI_006	2010		PRJEB2589
23	NEM316	1975	5	PRJNA334
26	CNCTC_10_84	1970	6	PRJNA229124
26	IT_NI_036	2010		PRJEB2589
26	IT_NI_037	2010		PRJEB2589
28	BG_NI_009	2008		PRJEB2589
61	SA111	2013		This work
88	DE_NI_012	2009		PRJEB2589
88	DK_NI_014	2009		PRJEB2589
88	DK_NI_016	2010		PRJEB2589
103	GBS85147	1995		PRJNA263907
103	M19	2010		PRJNA273437
103	SA172	2011		This work
110	2603V_R	1993		PRJNA330
144	BG_NI_001	2009		PRJEB2589
144	DE_NI_006	2008		PRJEB2589
196	DK_NI_019	2010		PRJEB2589
255	CZ_NI_007	2008		PRJEB2589
260	SA132	2011		This work
260	SA136	2011		This work
260	SA191	2011		This work
260	SA245	2013		This work
260	SA256	2013		This work
260	SA289	2013		This work

260	SA53	2007		This work
260	SA73	2008		This work
260	SA75	2008		This work
261	138p	2007		PRJNA226756
261	138SPAR	2011		PRJNA226756
261	2_22	1986		PRJEB643
261	GX026	2011		PRJNA254961
283	CU_GBS_08	2008		PRJNA274685
283	CU_GBS_98	1998		PRJNA274685
283	SG_M1	2015		PRJNA293392
297	NGBS357	2011		PRJNA293858
315	BE_NI_007	2010		PRJEB2589
452	NGBS572	2012		PRJNA246096
459	NGBS061	2010		PRJNA246096
479	CZ_NI_014	2009		PRJEB2589
609	ILRI005	2004		PRJEB1694
617	ILRI112	2002		PRJEB1774
739	FWL1402	2014		PRJNA323692
927	SA102	2010		This work
927	SA218	2012		This work
927	SA85	2010		This work
927	SA95	2010		This work
927	SA97	2010		This work
NT	ATCC13813	1949		PRJNA53057
NT	BE_NI_008	2010		PRJEB2589
NT	DK_NI_021	2009		PRJEB2589
NT	S25	2015		PRJNA323652
NT	SA01	2003		This work
NT	SA020	2006		PRJNA174852
NT	SA05	2003		This work
NT	SA09	2005		This work
NT	SA159	2011		This work
NT	SA16	2006		This work
NT	SA184	2011		This work
NT	SA195	2011		This work
NT	SA201	2012		This work
NT	SA209	2012		This work
NT	SA212	2012		This work
NT	SA220	2013		This work
NT	SA30	2006		This work
NT	SA330	2013		This work
NT	SA33	2006		This work
NT	SA333	2014		This work
NT	SA341	2014		This work
NT	SA343	2014		This work

NT	SA346	2014		This work
NT	SA374	2014		This work
NT	SA375	2014		This work
NT	SA623	2015		This work
NT	SA627	2015		This work
NT	SA79	2009		This work
NT	SA81	2009		This work
ND ST[2]	BG_NI_006	2009		PRJEB2589
ND ST[2]	DE_NI_003	2008		PRJEB2589
ND ST[2]	GB_NI_005	2009		PRJEB2589
ND ST[2]	Gottschalk_992B	2006	6	PRJNA86449
ND ST[2]	H002	2011		PRJNA258310

[1]BEAST configuration to allow variable date of isolation. It means, year of isolation more or less years of precision.

[2]ND ST: not defined ST.
